# THE REPLICATION EFFICIENCY OF DENGUE VIRUS SEROTYPE 1 ISOLATED FROM PATIENTS WITH DENGUE FEVER IN HUMAN HEPATOCYTE CELL LINES

**DOI:** 10.21010/Ajidv19i2.4

**Published:** 2025-04-07

**Authors:** GENI Lenggo, WIDODO Lovendo Ilham, SWESTIKAPUTRI Chrecentia Hanna, SYAHRURACHMAN Agus, DEWI Beti Ernawati

**Affiliations:** 1 Doctoral Program in Biomedical Science, Faculty of Medicine, Universitas Indonesia Jl. Salemba Raya No 6, Jakarta, 10430 Indonesia; 2 Magister Program in Biomedical Science, Faculty of Medicine, Universitas Indonesia Jl. Salemba Raya No 6, Jakarta, 10430 Indonesia; 3 Department of Microbiology School of Medicine and Health Sciences Catholic University of Indonesia Jl Pluit Raya Selatan 19 North Jakarta; 4 Department of Microbiology, Faculty of Medicine, Universitas Indonesia, Jl.Pegangsaan Timur 16, Jakarta 10320; 5 Study Program in Medical Laboratory Technology, Universitas Mohammad Husni Thamrin. Jl. Raya Pd. Gede No.23-25, Jakarta, 13550 Indonesia

**Keywords:** Huh-7, HepG2, Vero, DENV-1, Efficiency, Replication

## Abstract

**Background.:**

The Efficiency of viral replication in cells depends on the capability of supporting virus replication by the cells. We characterized the effectiveness of Dengue Virus Serotype 1 (DENV-1) replication in various cell lines and various multiplicity of infection (MOI) starting from 2 FFU/cell up to 0,3125 FFU/cell.

**Materials and Method.:**

We used HepG2 and Huh-7 human hepatocyte cell lines and in addition, we also used non-human kidney cells (Vero cells). DENV-1 strain IDS 11/2010 was isolated from DF patients and previously propagated in Huh7 and Vero cells as DENV-1-adapted Huh-7 and DENV-1-adapted Vero cells, respectively. Huh7 cells, Hep G2 cells, and Vero cells were infected with DENV-1 at various MOI and incubated for 48 hours at 37^0^C with 5% CO_2_. DENV-infected cells were determined by indirect immuno-peroxidase staining using 3,3’-Diaminobenzidine (DAB). DENV-1 infected cells as foci were counted under inverted light microscopy and were used to determine the virus titer.

**Results.:**

The virus was adapted to Huh-7 and Vero cells, with results showing that Vero cells exhibited the highest replication efficiency, evidenced by significant viral titers. Among human hepatocyte cell lines, DENV-1 demonstrated greater replication in Huh-7 cells than in HepG2 cells. Notably, no foci formation was observed in HepG2 cells after 48 hours of infection.

**Conclusion.:**

These findings underscore the suitability of Vero and Huh-7 cells as optimal environments for DENV-1 replication, offering valuable insights for enhancing laboratory diagnostics and advancing antiviral strategies and vaccine development against DENV-1.

## Introduction

Dengue disease is of great importance to the global public health. DENV-1 infection was associated with manifest clinical diseases from mild to severe, and increased mortality related to organ involvement (Jácome *et al.*, 2021) A disturbance in the control of Dengue virus (DENV) and the mechanisms that regulate cytokine production can increase the viral load accompanied by excessive production of pro-inflammatory cytokines, resulting in severe Dengue disease. Up to now, there are no commercially available antiviral drugs to treat DENV infection, and treatment remains supportive (Obi *et al.*, 2021). DENV vaccines have recently been used in some countries, its indication is limited due to the risk of severe Dengue in certain populations and the decline in efficacy during the second year in 4–16-year-olds in dengue-endemic countries (López-Medina *et al.*, 2022). *In vitro*, cell line-based assays were needed to screen vaccines and antiviral activity against DENV (Panda *et al.*, 2021; Tsypysheva *et al.*, 2021).

The replication of DENV depends on the susceptibility of the cell and the characteristics of its innate immune response (Charretier *et al.*, 2018; Lu *et al.*, 2018; Haryanto *et al.*, 2019). DENV-1 has the highest viral growth among all serotypes (Sasmono *et al.*, 2015). Appropriate cell models are essential to advance research into the molecular basis of the disease, which can be applied to studies of drug metabolism, hepatotoxicity, and even viral infections. Non-human (Vero cells) and Human hepatoma (Hep G2 cells and Huh-7 cells) are suitable for determining susceptibility to DENV infection (Chan *et al.*, 2016). Vero cells, HepG2 cells, and Huh-7 cells are readily available and easy to maintain, and they are commonly used for DENV replication, NS1 secretion, and immune responses (Kongmanas *et al.*, 2020).

DENV manipulates the host cell machinery to facilitate its replication during infection. DENV infects the host cell to release three structural proteins such as envelope (E), membrane (M), and capsid (C)) and seven nonstructural proteins (NS1, NS2a, NS2b, NS3, NS4a, NS4b, and NS5). It is known that the E protein is the molecule that binds to receptors on the host cell membrane and is also a major antigen, which can induce neutralizing antibody and host-specific protective immunity (Chin *et al.*, 2007). The receptors used by DENV may differ very much, depending on cell types and viral serotypes (Sakoonwatanyoo *et al.*, 2006). Heparan sulfate proteoglycans (HSPGs) and Heparan sulfate are major DENV receptors in the liver cells that play a role in the initial DENV binding of HepG2 cells, and Huh 7 cells (Fang *et al.*, 2013; Kongmanas *et al.*, 2020).

The expression levels of those receptors on HepG2 cells might be different from those of Huh-7. Furthermore, DENV replication in HepG2 cells was promoted by miR-21 (Clark *et al.*, 2016). Besides E protein, Non-structural proteins play an important role in virus adaptation to cultured cells. RACK1 is a host factor in HepG2 and Huh7 cells that interacts with the DENV-1 NS1 protein (Shue *et al.*, 2021). The efficiency of viral replication in cells depends on the differential cell susceptibility. Releasing DENV from host alternation facilitates adaptation. A fitness increase in one host usually diminishes fitness in another. Viruses allowed to specialize in single host cells exhibited fitness gains, which affects the replication of the virus. DENV fitness can be influenced by the cell lines it passes through. The virus was inoculated into two cell lines alternately, showing increased DENV fitness in both cell lines. Comparison of fitness and the general pattern of DENV-1 adaptation in differential cell lines affecting the efficiency of viral propagation (Vasilakis *et al.*, 2009; Scroggs *et al.*, 2021).

This study aims to compare the effectiveness of DENV-1 replication in different cell lines, human hepatocytes (Huh-7, HepG2), and non-human kidney cells (Vero). We used DENV-1 isolated from patients that were propagated in 2 cell lines (Huh-7 cell and Vero cell) with no significant difference in their titer.

The effectiveness of virus replication was measured based on the virus titer obtained from DENV-1 infected Huh-7, HepG2, and Vero with various multiplicity of infection (MOI) starting from 2 FFU/cell up to 0.03125 FFU/cell. Viral titration was based on the formation of foci on cells stained by indirect immuno-peroxidase staining with 3,3’-Diaminobenzidine (DAB). The result of this study is the basis for selecting suitable adapted DENV and cell lines for further studies such as the pathogenesis of DHF, diagnostic kit, antiviral drugs, and vaccines.

## Materials and Methods

### Viral isolates and cell culture

We used Dengue Virus Serotype1 strain IDS 11/2010 which was isolated from patients, and propagated in Vero cells and Huh7 cells, as working stocks of the viruses. Human cell lines were human hepatocyte Huh-7D12 (catalog No. 01042712, ECACC, England) and HepG2 (from LIPI Chemical Research Centre) were inoculated in DMEM with 10% FBS and 1% Penstrep (catalog No.15140122, Gibco™, United States). A non-human kidney cell line Vero (E6C1008 was obtained from the Indonesian Ministry of Health Research and Development Agency) was grown in MEM (catalog No.11095080, Gibco™, United States) supplemented with 10% FBS with 1% Penstrep. All culture media for cell lines were supplemented with 2 mM L-glutamine (Sigma-Aldrich).

### Ethical Considerations

Before the study, ethical approval was obtained from the FKUI-RSCM Health Research Ethics Committee on July 14, 2022 (Approval No. Ket-828/UN2. F1/ETIK/PPM.00.02/2022). The study was explained to the participants, and only those who provided written informed consent were included. Confidentiality and privacy was maintained throughout the study

### Cell lines propagation

The stock cells from the cryo tube were thawed quickly, immediately put into a 15 ml Falcon tube, and added DMEM (D6429-500 ML, Sigma-Aldrich, Munich, Germany) with 10 % FBS for Huh7 and HepG2, MEM 10 % FBS for Vero. Then centrifuged at 1200 rpm for 5 minutes. The pellets were re-suspended with DMEM or MEM 10% FBS (USA origin steril, F2442-500, Sigma-Aldrich, Munich, Germany) and put into Flask T25, then incubated at 37^o^C, 5% CO_2_. After the monolayer, cells were sub-cultured using new flasks.

### DENV-1 propagation

We used two types of DENV-1; DENV-1 was propagated in Vero cells and Huh-7 cells. DENV-1 was propagated in Vero cells and named DENV-1 adapted Vero cells, and in Huh-7 cells as DENV-1 Huh-7-adapted. The medium of monolayer cells in Flask T 25 was removed, and then 500 ul of DENV-1 virus stock isolated from patients and propagated in C6/36 for less than 5 passages was added. The flask was incubated for 2 hours at 37^o^C, 5% CO_2_, agitated every 30 minutes, and then 8 ml of DMEM/ MEM medium with 10% FBS was added. The flask was incubated at 37^o^C, 5% CO_2_. Every day the cytopathic effect (CPE) was observed. Supernatants were harvested on the 4^th^ or 5^th^ day post-infection (Dewi *et al.*, 2018). The supernatant was centrifuged at 1000 rpm for 5 minutes at 4^o^C to remove cell debris. Then, aliquot viral culture supernatant was transfered into 1 ml Eppendorf tube, and stored at -80^o^C, subsequently a titration was carried out to determine the viral titer. In this study, we used DENV-1-adapted Vero and Huh-7 cells.

### DENV-1 titration using the focus assays method

Cells were cultured in a 96-well plate and incubated for 24 hours at 37°C with 5% CO_2_. DENV-1 adapted Vero and Huh-7 cells, serially diluted (10^-1^ to 10^-4^), were added to the culture and incubated for 2 hours at 37°C with 5% CO_2_, agitated every 30 minutes. After removing the supernatant, DMEM/MEM 2% FBS medium was added, and incubation was continued for 48 hours at 37°C with 5% CO_2_. The infected supernatant was transferred to new cells in a 96-well plate and incubated for 2 hours at 37°C with 5% CO_2_ agitated every 30 minutes. The supernatant was discarded, and 1% methylcellulose in MEM 2% was added, followed by incubation for 48 hours at 37°C with 5% CO_2_, then fixed with 3.7% formaldehyde for focus assays were performed to determine the virus titers. After fixation and washing with PBS, the cells were added with Triton-X 0.5% to increase cell permeability. Then, human IgG anti-dengue from DENV-infected patients was added as the primary antibody. After 1 hour of incubation, cells were washed with PBS and incubated with horseradish peroxidase (HRP) labelled Goat anti-human IgG for 1 hour. After PBS washing, cells were treated with diaminobenzidine (DAB) substrate (diaminobenzidine, peroxidase staining kit) and observed under an inverted light microscope. The microscopic fields were chosen and foci cells were counted. DENV-1-infected cells appeared as brownish foci (Angelina *et al.*, 2017).

### DENV-1 infection in various cell lines

**Figure 1:**
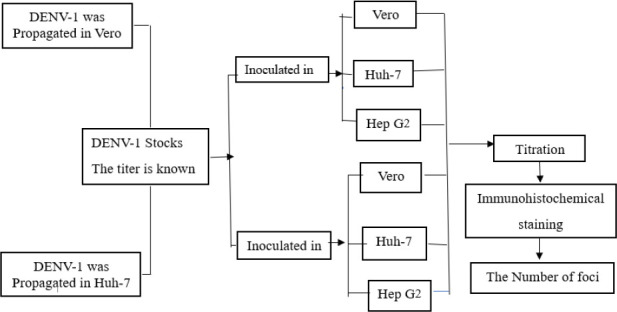
DENV-1 Propagation and Titration Scheme. DENV-1 was previously propagated on two cell lines, Vero and Huh-7 cells to obtain 2 viral stocks. The two virus stocks were inoculated into three cell lines: Vero, Huh-7, and HepG2. Furthermore, a titration was carried out to determine the viral titer obtained. The virus concentration inoculated in each cell line was measured using Immunohistochemical staining based on the number of foci in dilution series multiplicity of infection (MOI) from 2 FFU/cell up to 0,03125 FFU/cell.

The cell lines (Huh-7, HepG2, Vero) were subsequently cultured in 96 plates incubated at 37^o^C with 5% CO_2_ for 24 hours. DENV-1 adapted Huh-7 and Vero cells, at various multiplicity of infection (MOI) from 2 FFU/cell up to 0,03125 FFU/cell were inoculated into monolayer cells in each plate and incubated at 37^o^C, 5% CO_2_ for 2 hours, agitated every 30 minutes. After incubation, the cell supernatant was discarded and DMEM/MEM 2% FBS medium was added and incubated at 37^o^C with 5% CO_2_ for 48 hours. The three new cell lines in a 96-well plate, after a 24-hour incubation at 37^o^C with 5% CO_2._were exposed to supernatant from DENV-1-infected cell lines. The plates were incubated for 2 hours at 37^o^C, 5% CO_2_ with agitation every 30 minutes. Following this, the supernatant was removed and 1% methylcellulose in MEM 2% FBS was added. The plates were then incubated for 48 hours, and fixation with 3.7% formaldehyde for subsequent focus assays. The summary and flow of this study are explained in Figure 1.

## Result

**Figure 2:**
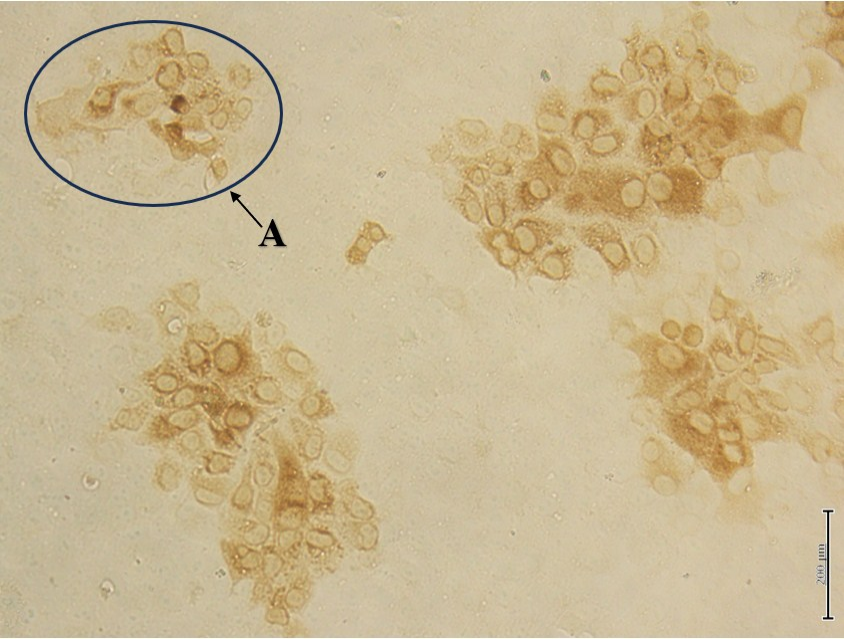
Characteristic foci in Vero cells at 48 hours post-infection. (HPI). The image shows clusters of cells exhibiting characteristic foci formation, indicated by the brown staining. The foci are marked by arrow A (circled area) and represent areas of interest where morphological changes consistent with transformation or infection are visible. The scale bar represents 200 µm. Foci were observed under inverted microscopy (original magnification ×400). (A) Foci in infected cells.

**Figure 3:**
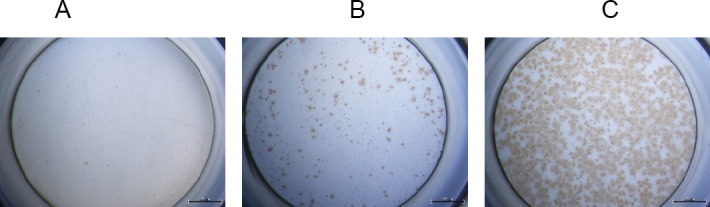
Results of immunostaining on DENV-1 infected Vero cell. The panels showed various formatted foci of DENV-1. Panel A displays the uninfected control cells, which showed no foci with brown color. Panel B illustrates the countable foci of DENV-1 infected cells with scattered foci. Panel C showed focus assay results with uncountable (NC) foci. The scale bars represent 200 µm. Foci were observed under inverted microscopy (original magnification ×400).

The effectivity of DENV-1 replication in cells was observed within variant cell lines as defined by the presence of foci from the focus assay. One focus with brown color was representative of one DENV-1 (Figure 2.). The formation of foci on cells infected with DENV-1 compared to cells not infected with DENV-1 as a control. Figure 3 is an example of a DENV-1 infected cell with various conditions of foci starting from uninfected, countable foci up to uncountable foci. We used Vero cells to represent various conditions of foci.

### DENV-1 adapted Huh-7 cells in various cell lines

Viral interference with the cell cycle can have a myriad of different effects to improve virus infection. We used DENV-1-adapted Huh-7 and DENV-1-adapted Vero cell lines to determine the effectiveness of DENV-1 replication in various cell lines. Various cell lines were infected with DENV-1 adapted Huh-7 at various MOI starting from 2 FFU/cell up to 0,03125. As a negative control, we used culture media of the cell. Table 1 showed that HepG2 cells were not infected by DENV-1-adapted Huh-7. We found that the number of foci was increased in the MOI dependent manner. Infection with an MOI of 0,0625 FFU/cell caused uncountable foci in Vero cells. However, we found 80 ± 4,34 foci of DENV-1 in Huh-7 cells. When we compared DENV infectivity between Vero cells and Huh-7 cells, we found that DENV-1 adapted Huh-7 more profound in Vero cells than Huh-7 cells (Table 1).

**Table 1:** Foci of DENV-1 adapted Huh-7 cells in variant cell lines at 48 hours post-infection.

		The Number of Foci (Mean ± SD)	

	Huh-7 (Human liver cell)	HepG2 (Human liver cell)	Vero (Monkey kidney cell)
MOI (FFU/cell)			
2	NC*	ND*	NC
1	NC	ND	NC
0,5	NC	ND	NC
0,25	NC	ND	NC
0,125	NC	ND	NC
0,0625	80 ± 4,34	ND	NC
0,03125	63 ± 9,70	ND	384 ± 4,44

* NC: Not Countable, ND: Not Detectable

### DENV-1 adapted Vero cells in various cell lines

The susceptibility of various cell lines to DENV-1-adapted Vero cells showed a similar trend with DENV-1-adapted Huh-7 cells. Huh-7 and Vero cells were highly susceptible to DENV-1-adapted Vero cells. Infection with an MOI of 0,0625 FFU/cell and above, showed uncountable foci due to a huge number of cells infected by DENV-1 adapted Vero cells (Table 2.). HepG2 cells were not susceptible to both DENV-1-adapted Vero and Huh-7 cells.

**Table 2:** Foci of DENV-1 adapted in Vero cells in variant cell lines at 48 hours post-infection.

		The Number of Foci (Mean ± SD)	

	Huh-7 (Human liver cell)	Hep G2 (Human liver cell)	Vero (Monkey kidney cell)
MOI (FFU/cell)			
2	NC*	ND*	NC
1	NC	ND	NC
0,5	NC	ND	NC
0,25	NC	ND	NC
0,125	NC	ND	NC
0,0625	NC	ND	NC
0,03125	99 ± 9,5	ND	117 ± 2,49

* NC: Not Countable, ND: Not Detectable

### Titer of DENV-1 adapted Huh-7 and Vero cells in various cell lines.

**Figure 4:**
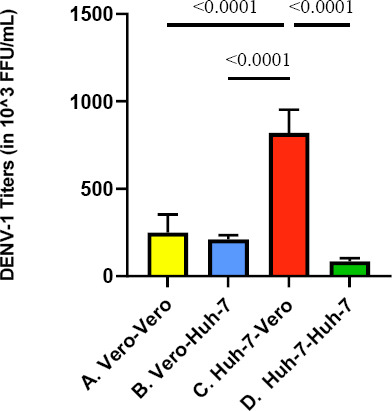
DENV-1 titers in variant cell lines 48 hours post-infection. DENV-1 was previously propagated in Huh-7 and Vero cells at an MOI of 0,03125 FFU/cell. A: Titer of DENV-1 adapted Vero cells in the Vero cells. B. Titer of DENV-1adapted Vero cells in the Huh-7 cells. C. Titer of DENV-1 adapted Huh-7 in Vero cells. D. Titer of DENV-1 adapted Huh-7 in Huh-7 cells. The graph shows the virus titers (in 10^3 FFU/mL)

The number of foci in the cell lines infected with various MOI of DENV-1 adapted Huh-7 or Vero was used to determine the virus titer. The higher the number of foci, the higher the virus titer. In Figure 4. it is known that the DENV-1 titers which were previously propagated in Huh-7 and Vero cells, were higher in Vero cells than in Huh-7 cells. The highest viral titer was found in DENV-1, adapted in Huh-7 cells, then inoculated on Vero cells. We calculated the titer from Huh-7 and Vero cells infected by DENV-1 adapted Huh-7 and Vero at a MOI of 0,03125 FFU/cell. We found that the average titer of DENV-1 adapted Huh-7 cells in Huh-7 and Vero cells was 85±17.70 (in 10^3 FFU/mL) and 818.8± 134.0 (in 10^3 FFU/mL), respectively. Based on the ANOVA test, there is a significant difference in the average DENV-1 titer adapted to Huh-7 and inoculated on Vero cells, compared to the average titer of DENV-1 adapted to Huh-7 and inoculated on Huh-7 cells, as well as the average titer of DENV-1 adapted to Vero cells and inoculated on both Vero and Huh-7 cells (Figure 4.).

## Discussion

The *Aedes Aegypti* mosquito is a vector for transmitting Dengue disease. DENV enters the human body through mosquito bites, penetrating the keratocyte layer and capillary network. Autopsies of human tissue from dengue fever patients showed that DENV replication affected the lymph nodes, spleen, alveolar macrophages in the lungs, and perivascular cells in the brain (Balsitis *et al.*, 2009). Studies conducted on Dengue Fever (DF) patients revealed that various types of cells such as monocytes, lymphocytes, macrophages, dendritic cells (DC), and endothelial cells can be infected with DENV (Durbin *et al.*, 2008). This is related to the severity of the disease (Durbin *et al.*, 2008). *In vitro*, many primary human cell types, including epithelial, endothelial (Dewi *et al.*, 2008), and fibroblasts have been shown to support viral replication (Marianneau *et al.*, 1996) Currently, various cell lines are widely used for the propagation of viral strains, antiviral studies, diagnostic tests, virus receptors, and replication studies using laboratory-adapted dengue virus strains. Low passages and genotypes from different geographic areas, marked variation in the susceptibility to infection among cell types (Schneider-Schaulies *et al.*, 2000) DENV infection in peripheral blood monocytes, U937 myeloid cells, and THP-1, result in increased antibodies (Diamond *et al.*, 2000). This study compared the replication effectiveness of DENV-1 isolated from DF patients in Indonesia in Human hepatocytes (Huh-7, HepG2) and non-human kidney cells (Vero). We used DENV-1-adapted Huh-7 cells and DENV-1-adapted Vero cells. Previous studies identified non-specific receptor molecules such as glycosaminoglycans and heparan sulfate, which function in DENV attachment to several cell lines (Chen *et al.*, 1997). Heparan sulfate consists of alternating hexuronic acid/D-glucosamine disaccharides, with different degrees and patterns of sulfation. This compound is expressed in almost all cell types and can form linear chains of varying structural complexity and length (Coombe and Kett, 2005). A principal role of the dengue virus envelope protein in the initial binding to target cells, especially the domain III, and detects amino acids 284-310 and amino acids 386-411 which are two potential heparan sulfate binding motifs in this domain (Coombe and Kett, 2005)

*In vivo* and *in vitro* studies show the involvement of the liver during dengue virus infections (Couvelard *et al.*, 1999) and the presence of the Dengue virus in hepatocytes and/or Kupffer cells (Huerre *et al.*, 2001). In the hepatocyte cell line, several stress-related proteins have been implicated as dengue virus receptors in various cell types, such as the GRP78 protein expressed in liver cells, and the HSP 90/70 protein in monocytes and macrophages (Jindadamrongwech and Smith, 2004). However, antibodies directed against hsp70 and hsp90 showed no inhibition of any Dengue serotype. The HSP 90/70 protein complex does not play a role in the internalization of the Dengue virus into liver cells, while the GRP78 protein is a Dengue virus receptor protein. This was confirmed by the presence of antibodies against GRP78 in the process of moderate inhibition of the entry of Dengue virus into liver cells (Cabrera-Hernandez *et al.*, 2007). We found DENV-1-positive infected cells (brown foci) in Huh-7 cells and Vero cells but not in Hep G2 cells. Previous studies showed that All four DENV serotypes can be propagated in HepG2 cells. Mature virus for serotype DENV-4 is produced within 12 hours, and for other serotypes is made at 17-18 hours (Thepparit *et al.*, 2004). However, pretreatment with the addition of trypsin or heparinase III to HepG2 cell cultures can cause a decrease in virus production, with the smallest effect on serotype DENV-3 (Thepparit *et al.*, 2004). Pretreating HepG2 with trypsin might reduce the ability of DENV-1 to infect HepG2 in this study.

Recent studies have focused on the entry of the DENV into HepG2 cells, identifying potential receptors or factors crucial to this process. The study identified the 37-kDa/67-kDa laminin receptor as a DENV-1 receptor in HepG2 cells, demonstrating its role in virus entry, with reduced binding observed after pre-incubation with soluble laminin and an antibody against GRP78 (Ng *et al.*, 2022). Another study investigated the mechanism of Dengue virus internalization into HepG2 cells and found that the entry was independent of hsp90 and hsp70, which are heat shock proteins that have been implicated in the entry of DENV into other cell types (Wang *et al.*, 2020). Furthermore, in a study, the simian kidney cell line Vero had higher susceptibility to DENV-1 than the human hepatoma cell line HepG2. This means that the infectivity and binding affinity of DENV-1 is higher in Vero cells than in HepG2 cells. The study suggested the involvement of different receptors or receptors presented in a different conformation in the two cell types.(Thepparit *et al.*, 2013). Therefore, in this receptor mechanism, there may be differences in the receptors or their conformation between HepG2 and Vero cells.

DENV uses heparan sulfate (HS) on the surface of Vero cells as a receptor (Kongmanas *et al.*, 2020). Vero cells are highly susceptible to dengue virus infection, and they contain large amounts of heparan sulfate (HS) on their surfaces, which serve as receptors for the virus1. In addition to HS, other molecules have been identified as putative Dengue virus receptors on Vero cells, such as a 74-kDa protein and glycoproteins of 40 and 45 kDa. The 74-kDa protein has been suggested to be part of a putative receptor complex for Dengue virus in Vero cells (Rabelo *et al.*, 2017). The entry of Dengue virus into Vero cells involves clathrin-mediated endocytosis, which plays a role in the outcome of infections. The DENV-3 infective entry pathway used varies depending on the host cell. The influence of the clathrin pathway on DENV-3 entry has been evaluated in other mammalian cell lines, such as HepG2, U937, and A549 cells.

Infectious and non-infectious pathways experience simultaneous coexistence depending on the usage of clathrin-mediated endocytosis has been demonstrated for the first time in Vero cells2 (Carpp *et al.*, 2020). In addition to Vero cells, other cell lines like iPS-ML and iPS-DC have been studied for their susceptibility to DENV infection. Vero cells, K562, iPS-DC, and iPS-ML, are susceptible to DENV infection. The highest infective yield titer was observed in Vero cells (Bolívar-Marin *et al.*, 2022). Huh-7 cells are susceptible to infection by all four Dengue virus serotypes, with research identifying a putative receptor for DENV-1 on these cells (Zandi *et al.*, 2012). Additionally, a proteomic analysis of Huh-7 cells infected with DENV-2 revealed significant alterations in the expression of host cell proteins, providing insights into the interactions between the virus and the host cell (Bolívar-Marin *et al.*, 2022). The exact nature of the receptor on Huh-7 cells that mediates DENV entry has not been fully elucidated. However, the susceptibility of Huh-7 cells to infection by all four dengue virus serotypes suggests that they may express a receptor or receptors that are broadly recognized by the virus. DENV-1-adapted in Huh-7 cells and Vero cells can efficiently replicate in Huh-7 and Vero cells up to the MOI 0,03125 FFU/cell. In comparison with Huh7, the number of foci in Vero cells were more profound in both DENV-1-derived Huh-7 and Vero cells. DENV can manipulate the host cell’s machinery to facilitate its replication.

The processes that occur during DENV infection have potential in the study of the pathogenesis, vaccine, diagnostic, and therapeutic agents (Suttitheptumrong *et al.*, 2013). In the absence of an animal model, there had been hampering difficulties associated with studies of Dengue virus infection of the human liver, an appropriate hepatocyte model is essential to conduct this study. Huh7 and HepG2 cells are human hepatoma cell lines, which can be used as liver cell models. These cells are readily available and easy to maintain (Haryanto *et al.*, 2019; Jácome *et al.*, 2021). However, such carcinoma-derived cell lines have genetic and characteristic limitations. The hepatic model can be applied to studies of drug metabolism, hepatotoxicity, and even viral infections. Heparan sulfate has previously been shown to be a major DENV receptor in human liver cells (Gutiérrez-Barbosa *et al.*, 2020). With the limitation of Huh7 cell lines, we found that Huh7 can be infected with DENV-1 from DF patients in Indonesia-derived Huh-7 and Vero cells. Our findings revealed that efficiently replicating DENV is influenced by the type of cell lines used for propagation associated with the receptors present in the cell.

## Conclusion

The study demonstrated that Dengue Virus Serotype 1 (DENV-1), particularly the MOI 0,03125 (FFU/cell), exhibits different replication efficiencies in various cell lines. The virus adapted to both Huh-7 and Vero cells showed the highest replication efficiency in Vero cells, with significant viral titers observed. Among human hepatocyte cell lines, DENV-1 replicated more effectively in Huh-7 cells compared to HepG2 cells. This suggests that Vero cells and Huh-7 cells are more conducive environments for DENV-1 replication than HepG2 cells, which did not exhibit foci formation after 48 hours of infection. These findings are crucial for optimizing laboratory diagnostics and developing antiviral strategies and vaccines targeting DENV-1.

## Conflict of Interest

The authors declare that there are no conflicts of interest associated with this study.

**List of Abbreviations:**
DENV-1-: Dengue Virus Serotype 1;
HepG2-: Hepatocellular Carcinoma, G2;
Huh-7-: Human Hepatoma-7;
Vero-: Vervet Monkey Kidney;
MOI-: Multiplicity of Infection;
FFU-: Focus-Forming Units.
